# First person – Jeffry Jeve

**DOI:** 10.1242/bio.062940

**Published:** 2026-10-22

## Abstract

First Person is a series of interviews with the first authors of a selection of papers published in Biology Open, helping researchers promote themselves alongside their papers. Jeffry Jeve is first author on ‘Early life stages of stress-tolerant corals (*Favites* spp.) exhibit tolerance to acute thermal stress’, published in BiO. Jeffry is a research assistant in the lab of Maria Vanessa Baria-Rodriguez at the University of the Philippines Diliman, Quezon City, Philippines, investigating coral biology and ecology, specifically looking at early life histories and how environmental stressors affect them.

**Figure:**
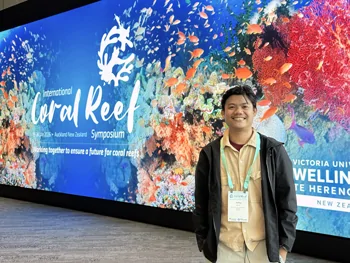
Jeffry Jeve

**Describe your scientific journey and your current research focus**

My scientific journey began during my high school science years, where I first engaged in experimental research exploring if organic waste fertilisers like eggshells and banana peelings could enhance mung beans' growth and development. That early experience taught me the fundamentals of the scientific method and sparked my interest in research. This foundation was strengthened during my undergraduate years at Visayas State University (VSU), Baybay, Philippines, where my thesis focused on planktonic community assessment, shifting my interest toward marine ecosystems. Following my undergraduate degree and a brief teaching stint at VSU, I joined the Interactions of Marine Bionts and Benthic Ecosystems Laboratory at the Marine Science Institute, University of the Philippines Diliman. Within this lab, my work focused on coral biology and ecology, including the impacts of relevant environmental stressors. Being based at the Bolinao Marine Laboratory, my interest in scientific research naturally progressed into broader ecological and reproductive studies of corals. Currently, my research focuses on how coral early life stages respond to heat stress and their early post-settlement responses to turbid and degraded reefs. Looking back, my journey has been a series of connected paths, with each step building on the last, driven by a continuous curiosity and support from family, friends, mentors, and colleagues.

**Who or what inspired you to become a scientist?**

Growing up, I never actually had a master plan or dream to become a scientist. Honestly, even today, the title feels quite big to carry. What drew me into this field wasn't a lifelong ambition, but rather an enjoyment of the scientific method, particularly the thrill of designing an experiment and seeing it through. Perhaps it is also because I feel a sense of emptiness when I do not have a project lined up, whether that means an experiment to design, data to analyse, or a paper to write, which is what I simply call ‘work’. That enjoyment of pursuing research was completely shaped by the mentors and colleagues I've worked with, largely due to the inspiring environments I was lucky enough to be in. Witnessing their passion for science inspired me, prompting me to channel my own energy into creating meaningful scientific knowledge for the future.

**How would you explain the main finding of your paper?**

What surprises a lot of people I know is that corals start life as soft, tiny fragile babies before anchoring down to build hard reefs. Because they are so delicate during early life stages, sudden spikes in seawater temperature can be dangerous for them. Our research tested a particularly tough species of hard coral and we found good news: these specific babies could survive short, intense heat stress in their earliest days. However, we only looked at their immediate survival over a short period, and we did not look inside their bodies to see the deeper, hidden costs that heat stress might take on their internal health. While different coral species have different tolerance levels depending on their parents and environment, they are all ultimately facing a difficult future due to a rapidly changing climate.

**What are the potential implications of this finding for your field of research?**

The primary implication of this work is that it introduces a practical framework for identifying climate-resilient corals at their most vulnerable life stage. By serving as an early thermal screening tool, this approach can help researchers quickly pinpoint species capable of tolerating intense heat shocks. To truly maximise its impact on coral rehabilitation, these laboratory insights now need to be coupled with field validations to ensure high settlement and survival rates in the natural setting. While this provides valuable baseline support for evolving active reef restoration, it also opens up an essential research avenue to examine whether this short-term heat tolerance comes with hidden long-term ecological or physiological trade-offs.

**Figure:**
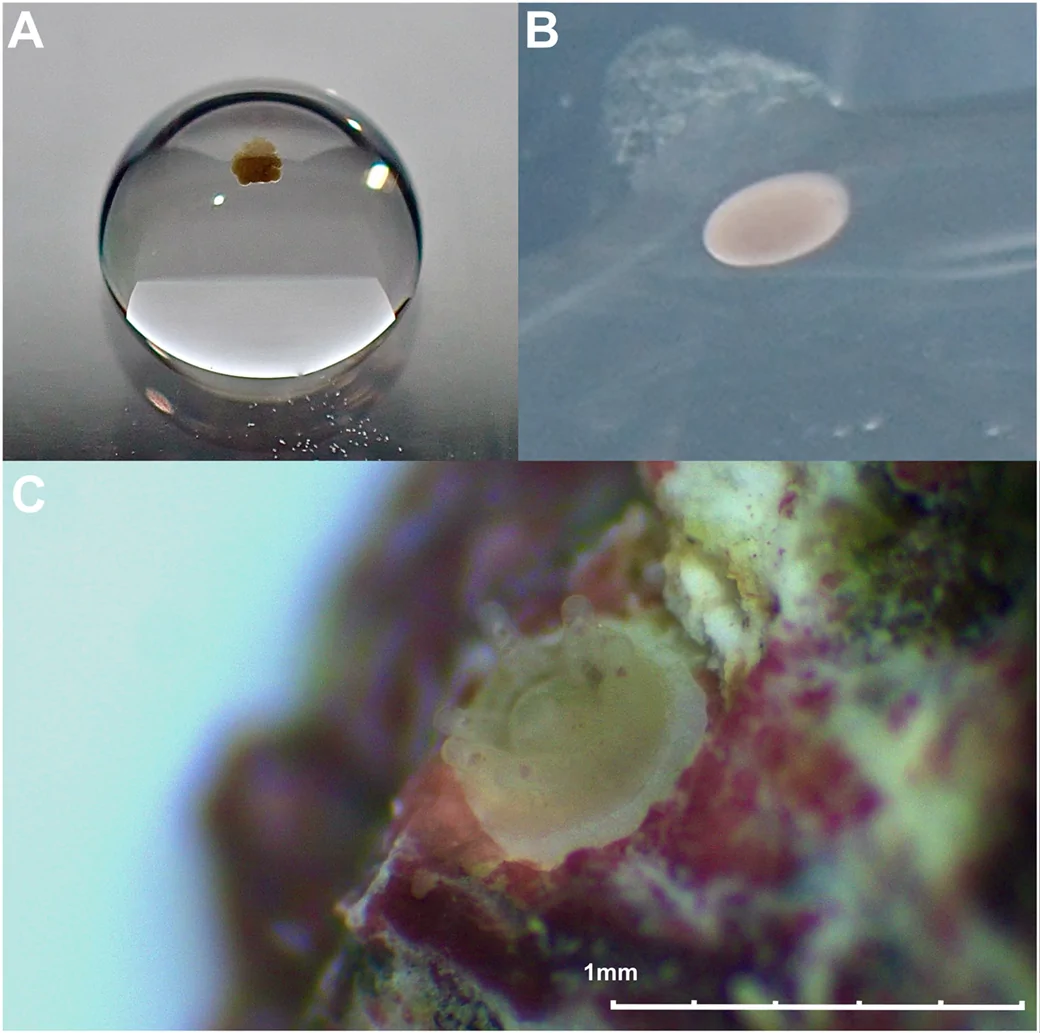
**Representative photos of coral early life stages.** (A) A developing embryo suspended in a seawater droplet, (B) a motile larva stage, and (C) a 1-week-old coral recruit post-settlement. Photos A and B were captured using an OM System Tough TG-7 camera, while photo C was documented under a Motic SMZ-171 stereomicroscope.

**Which part of this research project was the most rewarding?**

At first, I thought the most rewarding part was presenting our findings at conferences and connecting with peers. However, when our paper was officially accepted for publication, it brought a completely different sense of triumph, as my first lead-author paper. Seeing the research move all the way from the initial planning and intense laboratory phases to a peer-reviewed journal was incredibly fulfilling. It made every hour of hard work feel completely validated, knowing that we have successfully contributed a new and reliable knowledge to the scientific community.

**What do you enjoy most about being an early-career researcher?**

What I enjoy most about being an early-career researcher is the opportunity to actively build upon the work I have already started. I think right now is the perfect time for me to go beyond my current scope by learning advanced tools and methodologies, as well as exploring different marine taxa to support what I have already been working on. It is an exciting phase because I am open to new opportunities while simultaneously building the foundational portfolio and track record needed to secure PhD opportunities and future research projects. Furthermore, I really value this period as a chance to expand my professional network, connecting with mentors, fellow researchers, and collaborators who share the same discipline and passions.

**What piece of advice would you give to the next generation of researchers?**

From my early-career experiences, my biggest piece of advice would be to not give up easily when things go wrong. In science, I think it is rare for our experiments or field plans to work out exactly the way we want on the first try. However, I have learned that unexpected setbacks often lead to something much more meaningful and rewarding, pushing us to ask better questions or test how flexible we can be with the resources we have, without compromising the outcome of our studies. There is a certain timing to research; sometimes a failed trial simply redirects you toward a better discovery and unexpected opportunities. This reminds me of a quote a close friend always brings up, which is that opportunities are prioritised for those who are prepared. I think resilience is just as important as curiosity, and if a path is meant for you, your persistence and preparation will eventually bring you to it.

**What's next for you?**

Moving forward, my immediate goal is to continue doing research while actively applying for PhD opportunities that align with my field. Specifically, I want to expand from organismal ecophysiology into broader coral reef ecology, with a particular focus on stress ecology and reproductive ecology. I think this transition will allow me to connect the coral early life histories I've been studying in the lab to large-scale reef dynamics and active reef conservation and restoration. My aim is to further my studies where I can learn advanced tools and methods to support this next phase of my research career.
